# Circulating Tumor Cells as an Indicator of Treatment Options for Hepatocellular Carcinoma Less Than or Equal to 3 cm in Size: A Multi-Center, Retrospective Study

**DOI:** 10.3389/fsurg.2022.895426

**Published:** 2022-06-20

**Authors:** Qiao Zhang, Feng Xia, Hengyi Gao, Zhenheng Wu, Wenjing Cao, Qingfeng Xiang, Zhifeng Guan, Yang Su, Weiqiao Zhang, Weiqiang Chen, Ali Mo, Shuqun Li

**Affiliations:** ^1^ Department of Hepatobiliary Surgery, Zhongshan Hospital Affiliated to Sun Yat-Sen University, Zhongshan, China; ^2^ Department of Hepatic Surgery, Tongji Hospital of Tongji Medical College of Huazhong University of Science and Technology, Wuhan, China; ^3^ Department of Hepatic Vascular Surgery, Xiaogan Central Hospital, Xiaogan, China; ^4^ Department of Hepatobiliary Surgery, Union Hospital Affiliated to Fujian Medical University, Fuzhou, China; ^5^ Southern Medical University Graduate School, Guangzhou, China; ^6^ The First Department of General Surgery, Qingyuan People’s Hospital, Qingyuan, China; ^7^ Department of Gastrointestinal Surgery, Zhongshan Hospital Affiliated to Sun Yat-Sen University, Zhongshan, China; ^8^ Department of Hepatobiliary Surgery, Renmin Hospital of Wuhan University, Wuhan, China; ^9^ Guangdong Medical College, Zhanjiang, China; ^10^ Department of Hepatobiliary and Pancreatic Surgery, Affiliated Hospital of Guilin Medical University, Guilin, China

**Keywords:** hepatocellular carcinoma, circulating tumor cell, early recurrence, radiofrequency ablation, surgical resection

## Abstract

**Background:**

The status of circulating tumor cells (CTCs) is related to the recurrence of hepatocellular carcinoma (HCC), which is also one of the reasons for the poor prognosis of HCC. The purpose of this study was to explore whether CTCs can help guide the choice of treatment methods for HCC.

**Methods:**

This study is a multicenter retrospective study, including 602 patients with HCC. CTCs were detected in the overall cohort before operation. There were 361 patients in the training cohort and 241 patients in the validation cohort. Patients were divided into CTC-negative group (CTCs = 0/5 mL) and the CTC-positive group (CTCs ≥ 1/5 mL) according to CTCs status. Subgroup analysis was performed according to CTCs status. We compared overall survival, and recurrence outcomes for HCC patients with different CTC statuses after undergoing radiofrequency ablation (RFA) or surgical resection (SR)

**Results:**

There was no significant difference in overall survival (OS) and recurrence-free survival (RFS) between the RFA group and SR group for CTC-negative patients in both the training cohort and the validation cohort (*P* > 0.05). However, among CTC-positive patients, the clinical outcome of patients in the SR group was significantly better than those in the RFA group. CTC-positive patients who underwent RFA had increased early recurrence compared to those who underwent SR. RFA is an independent risk factor for survival and recurrence in CTC-positive HCC patients

**Conclusions:**

The CTC status could serve as an indicator to guide the choice between surgical resection or radiofrequency ablation for early hepatocellular carcinoma. Surgical resection is recommended for CTC-positive patients.

## Introduction

Primary liver cancer (PLC) is a malignant tumor derived from human hepatocytes and bile duct epithelial cells, of which hepatocellular carcinoma is the most common, accounting for about 90% of PLC (1, 2). HCC is also the fourth leading cause of cancer-related death worldwide (3). In the past 10 years, with the improvement of people’s awareness of HCC surveillance and the level of diagnosis, more and more early stage HCC patients are detected in time (4). Surgical resection (SR) and liver transplantation (LT) have long been considered first-line treatment options for patients with early-stage HCC who can tolerate surgery (5), with a 5-year survival rate of around 70%–80% (1). However, in the clinical treatment of HCC, not only should the location of the tumor and, the reserve of liver function be put into consideration, but many other factors such as the family’s economic status and the patient’s willingness must also be considered, only about 20% of the patients are willing and suitable for surgery (6).

Percutaneous radiofrequency ablation (RFA), as one of the important components in the comprehensive treatment of early HCC, has the advantages of excellent clinical efficacy, less trauma, high safety, and reproducible application (7, 8). RFA plays an increasingly important role in the comprehensive treatment of early HCC. But, the appropriate population for RFA treatment of early-stage hepatocellular carcinoma has been controversial (9–13). Therefore, there is an urgent need for reliable indicators to guide the choice of RFA or SR in the treatment of early-stage HCC.

Previous studies have reported that preoperative CTCs status is associated with recurrence after RFA (14), and some studies have confirmed that CTCs status can be used as an indicator to guide the extent of surgical margins for HCC (15). Therefore, we wondered whether preoperative CTCs status could guide the application of RFA in HCC treatment.

This study utilized a multicenter database to stratify HCC patients based on their CTCs status and evaluate the different impact of treatment modalities on early recurrence and overall survival, to explore and verify the optimal treatment of early-stage HCC in different CTCs statuses.

## Materials and Methods

### Patient Enrollment

This study enrolled 602 patients with HCC who were admitted to the Department of Hepatobiliary Surgery of Zhongshan People’s Hospital and Xiaogan Central Hospital from January 2014 to December 2019. Inclusion criteria: (1). the age of the patient at the time of diagnosis of HCC is not less than 18 years; (2). The maximum diameter of the tumor ≤3 cm in imaging or pathology, and the number of tumors ≤3; (3). the Child-Pugh classification of liver function is grade A or B; (4). not receiving any form of treatment intervention before operation; (5). postoperative pathological or clinical diagnosis of HCC; (6). all patients who underwent surgical resection were treated with radical surgical resection (R0 resection); (7). complete clinical data. Patients who met any of the following criteria were excluded from the study cohort: (1). patients with cardiac, cerebral, and renal dysfunction; (2). a history of other malignant tumors; (3). the presence of portal vein, hepatic vein, and hepatic artery tumor thrombus; (4). adjacent organs or distant metastasis; (5). lost to follow-up.

When tumor recurrence occurred, such as: re-radiofrequency ablation, re-surgical resection, salvage liver transplantation, transcatheter arterial chemoembolization (TACE), molecular targeted therapy, immunotherapy,would be performed according to the patient’s wishes and the recurrence pattern. The diagnostic criteria for HCC were determined according to the European Society of Liver Diseases non-invasive diagnostic criteria for primary HCC (16). Patients admitted to the Zhongshan People’s hospital were used for the training cohort, while patients admitted to the Xiaogan Central Hospital were considered as the validation cohort. The study was approved by the Research Ethics Board of the Zhongshan People’s Hospital and Xiaogan Central Hospital, with written informed consent obtained from every patient. This study declares compliance with the norms of the Declaration of Helsinki.

### Data Collection

We collected information on demographic and clinicopathological characteristics of all patients, including age, gender, hepatitis B virus (HBV) and, hepatitis C virus (HCV) infection status, cirrhosis, Child-Pugh classification, maximum tumor diameter, number of tumors, etc. At the same time, continuous variables such as age, alanine aminotransferase (ALT), aspartate aminotransferase (AST), *γ*-glutamyltra nsferase (GGT), alkaline phosphatase (ALP), albumin (ALB), total bilirubin (TIBL), creatinine (CR), international normalized ratio (INR), alpha-fetoprotein (AFP) were transformed into binary variables according to the upper and lower limits of recognized critical values or normal values (14).

### Therapy Method

#### Surgical Resection

Each patient was evaluated preoperatively, and a corresponding surgical plan was formulated according to the patient’s tumor location, tumor size, and the relationship with the adjacent large blood vessels in the liver. Surgical resection is divided into laparoscopic hepatectomy and open hepatectomy. The scope of surgical resection includes: segmentectomy, and hemihepatectomy. The standard of surgical margin is to ensure that the surgical margin is ≥1 cm away from the tumor boundary according to the Guidelines for Diagnosis and Treatment of Primary Liver Cancer in China (2017 Edition) (17). The basic procedure of the operation is as follows: First, perform anesthesia and routine disinfection. After entering the abdominal cavity, first observe whether the tumor has distant metastasis to the adjacent organs, and use the corresponding instruments to separate the corresponding tissue structure. This is followed by intermittent occlusion of the first porta hepatis and selective blockade of the second porta hepatis if necessary. The corresponding hepatic segment is cut off according to the tumor location. After finding no residual tumor at the resection margin, blood flow stoppage is performed at the resection margins, the first and second hepatic hilum were released, and finally the abdomen is closed. Surgical resection procedures are performed by senior surgeons who are senior associate chief physicians or above.

#### Radiofrequency Ablation

Each medical center conducts radiofrequency ablation according to the medical devices and equipment of the unit. The patient is instructed to take a supine position, and then the corresponding parts of the tumor are disinfected, sterile towels are laid, and local anesthesia is given. Under the guidance of the ultrasound probe, the location of the tumor, and the position and direction of the needle insertion are determined. Next, the radiofrequency ablation needle is inserted into the lesion, and radiofrequency ablation is performed, and each ablation time is 6–8 min. For lesions with larger diameters, the needle can be inserted several times until the ablation area completely covers the tumor, and then the radiofrequency ablation needle is slowly pulled out. In general, the range of ablation should cover a 5-mm distance beyond the tumor boundary (17).

### CTC Isolation and Detection

Three days before surgery or RFA, we drew 5 mL of peripheral blood as a sample for inspection and strictly processed the sample according to the manufacturer’s instructions. The detection of CTCs was performed by the “Cyttel” method (Jiangsu, China), whose principles include the negative immunomagnetic particle method and immunofluorescence in situ hybridization (im-FISH) (18–20). The former mainly uses immunomagnetic particles (anti-CD45 antibody-conjugated magnetic beads) as the carrier, through the principle of antigen–antibody reaction, combined with centrifugation technology, to remove leukocytes from the blood in vitro to separate rare cells. Then, the samples were fixed on glass slides, dehydrated with ethanol, dried, and then hybridized with chromosome centromere probe No. 1 and chromosome centromere probe No. 8. Finally, 4-diamidine-2-phenylindole (DAPI) staining was added to seal the samples, and the CTCs were observed and counted under a fluorescence microscope (Microprofit, China) (14, 21). CTCs count ≥1/5 mL was defined as CTC-positive (22).

### Follow-Up

All patients were followed up through the outpatient service, telephone, or WeChat. Follow-up examination items included chest X-ray or chest CT scan, abdominal ultrasound, abdominal enhanced CT or MRI, and PET-CT. They were followed up every three months for two years after surgery, from the day of surgery, and every six months after two years after surgery. Overall survival (OS) was defined as the time from the date of surgery to the date of patient death or last follow-up, and Recurrence-free survival (RFS) was defined as the time from the date of surgery to the date of postoperative tumor recurrence or last follow-up. The last day of follow-up was December 1, 2021.

### Statistical Analysis

Continuous variables were expressed as median ± squared difference (Median ± SD), and categorical variables were expressed as the number of patients (n) or percentage (%). Continuous variables were compared by Student’s t-test or Mann-Whitney U test, and categorical variables were compared by *χ*^2^ or Fisher’s exact test. The survival curves of OS and RFS of patients were drawn by the Kaplan-Meier method, and the OS and RFS of the RFA group and SR the group were compared using the Log-rank method. We also used Landmark analysis to assess outcomes for early recurrence (≤24 months postoperatively) and late recurrence (>24 months). Univariate and multivariate Cox regression models were used to analyze the independent risk factors of related clinical variables for RFS and OS in patients. All statistics and graphics for this study were done in R language (version 3.62). A *P* value <0.05 was considered to be statistically significant.

## Results

### Patient Characteristics

In this study, a total of 602 HCC patients were included in the total cohort, with 361 patients in the training cohort and 241 in the validation cohort. The median follow-up time for the training cohort was 30.0 months (interquartile range, IQR 14.0–45.0 months) and the median follow-up time for the validation cohort was 31.0 months (interquartile range, IQR 15.0–44.0 months). In the training cohort, 146 (40.4%) had tumor recurrence and 84 (23.3%) died. In the validation cohort, 96 (39.8%) had tumor recurrence and 47 (19.5%) died. There were 147 (40.7%) and 99 (41.1%) CTC-positive cases in the training and validation the cohorts, respectively. Additional clinicopathological information is shown in Table 1, in the training and validation cohorts. There was no significant difference in clinicopathological information between the RFA group and the SR group (*P *> 0.05).

**Table 1:** Comparison of clinicopathological variables between Training cohort and Validation cohort.

Variable	Overall cohort (*n* = 602)		
	Training cohort (*n* = 361)	Validation cohort (*n* = 241)	*P*
Age (years)			
<60	120 (33.2)	76 (31.5)	0.727
≥60	241 (66.8)	165 (68.5)	
Gender			
Female	104 (28.8)	63 (26.1)	0.533
Male	257 (71.2)	178 (73.9)	
HBV			
No	36 (10.0)	23 (9.5)	0.973
Yes	325 (90.0)	218 (90.5)	
HCV			
No	348 (96.4)	230 (95.4)	0.704
Yes	13 (3.6)	11 (4.6)	
Cirrhosis			
No	94 (26.0)	54 (22.4)	0.359
Yes	267 (74.0)	187 (77.6)	
Child-Pugh			
A	321 (88.9)	217 (90.0)	0.762
B	40 (11.1)	24 (10.0)	
ALT (U/L)			
<50	299 (82.8)	189 (78.4)	0.213
≥50	62 (17.2)	52 (21.6)	
AST (U/L)			
<40	250 (69.3)	172 (71.4)	0.642
≥40	111 (30.7)	69 (28.6)	
GGT (U/L)			
<45	239 (66.2)	157 (65.1)	0.856
≥45	122 (33.8)	84 (34.9)	
ALP (U/L)			
<125	306 (84.8)	199 (82.6)	0.546
≥125	55 (15.2)	42 (17.4)	
Alb (g/L)			
<35	70 (19.4)	44 (18.3)	0.809
≥35	291 (80.6)	197 (81.7)	
TIBL (µmol/L)			
<20.4	281 (77.8)	182 (75.5)	0.573
≥20.4	80 (22.2)	59 (24.5)	
CR (µmol/L)			
<84	354 (98.1)	231 (95.9)	0.176
≥84	7 (1.9)	10 (4.1)	
INR			
<1.15	334 (92.5)	224 (92.9)	0.971
≥1.15	27 (7.5)	17 (7.1)	
AFP (µg/mL)			
<400	236 (65.4)	158 (65.6)	1.000
≥400	125 (34.6)	83 (34.4)	
Tumor diameter (cm)	23.24 (4.95)	24.21 (4.23)	0.053
Tumor number			
Single tumor	181 (50.1)	120 (49.8)	1.000
Multiple tumors	180 (49.9)	121 (50.2)	
Therapeutic method			
SR	215 (59.6)	147 (61.0)	0.788
RFA	146 (40.4)	94 (39.0)	
CTCs count			
0	214 (59.3)	142 (58.9)	0.998
≥1	147 (40.7)	99 (41.1)	

*AST, aspartate aminotransferase; ALT, alanine aminotransferase; GGT, gamma glutamyl transpeptidase; ALP, alkaline phosphatase; Alb, albumin; TIBL, total bilirubin; DIBL, direct bilirubin; CR, creatinine; INR, international normalized ratio; AFP, alpha fetoprotein; HBV, hepatitis B virus; HCV, hepatitis C virus; RFA, radiofrequency ablation; SR, surgical resection. CTC, circulating tumor cells.*

### Comparison of OS and RFS between the RFA and SR the Groups in the Training Cohort and the Validation Cohort

In the validation cohort, there were no statistically significant differences in the OS and RFS in the RFA and SR groups after the Kaplan-Meier analysis. (*P* > 0.05; Supplementary Figures S1B,D). In the training cohort, the RFS of the SR group was better than that of the RFA group, and the difference was statistically significant (*P *< 0.05; Supplementary Figure S1C), while in terms of OS, the efficacy of RFA and SR was almost the same, with no significant difference between the two groups (*P* > 0.05; Supplementary Figure S1A).

### Stratified Analysis by CTCs Status to Compare OS and RFS Between RFA and SR Groups

To explore whether CTCs status could guide surgical treatment modality, we stratified the training and validation cohorts according to CTCs status. The number of CTC-positive patients in the training cohort and the validation cohort was 147 (40.7%) and 99 (41.1%), respectively. In the CTC-positive group, 91 and 60 patients underwent SR in the training and validation cohorts, respectively. 56 and 39 patients underwent RFA in the training and validation cohorts, respectively. There were no significant differences in demographic and clinicopathological variables between the SR group and the RFA group in each subgroup (*P* > 0.05, Table 2). In the CTC-negative group, the SR and RFA groups also had no significant differences in demographic and clinicopathological variables in each subgroup, and there was no statistical difference (*P* > 0.05, Supplementary Table S1).

**Table 2:** Comparison of clinicopathological variables between SR and RFA in CTC-positive cohort.

Variable	Training cohort (*n* = 147)			Validation cohort (*n* = 99)		
	SR (*n* = 91)	RFA (*n* = 56)	*P*	SR (*n* = 60)	RFA (*n* = 39)	*P*
Age (years)						
<60	33 (36.3)	19 (33.9)	0.912	15 (25.0)	10 (25.6)	1.000
≥60	58 (63.7)	37 (66.1)		45 (75.0)	29 (74.4)	
Gender						
Female	27 (29.7)	13 (23.2)	0.507	14 (23.3)	16 (41.0)	0.099
Male	64 (70.3)	43 (76.8)		46 (76.7)	23 (59.0)	
HBV						
No	10 (11.0)	5 (8.9)	0.904	4 (6.7)	5 (12.8)	0.495
Yes	81 (89.0)	51 (91.1)		56 (93.3)	34 (87.2)	
HCV						
No	86 (94.5)	55 (98.2)	0.500	57 (95.0)	37 (94.9)	1.000
Yes	5 (5.5)	1 (1.8)		3 (5.0)	2 (5.1)	
Cirrhosis						
No	27 (29.7)	17 (30.4)	1.000	16 (26.7)	6 (15.4)	0.284
Yes	64 (70.3)	39 (69.6)		44 (73.3)	33 (84.6)	
Child-Pugh						
A	78 (85.7)	50 (89.3)	0.709	53 (88.3)	32 (82.1)	0.561
B	13 (14.3)	6 (10.7)		7 (11.7)	7 (17.9)	
ALT (U/L)						
<50	78 (85.7)	50 (89.3)	0.709	45 (75.0)	31 (79.5)	0.785
≥50	13 (14.3)	6 (10.7)		15 (25.0)	8 (20.5)	
AST (U/L)						
<40	68 (74.7)	33 (58.9)	0.068	44 (73.3)	27 (69.2)	0.830
≥40	23 (25.3)	23 (41.1)		16 (26.7)	12 (30.8)	
GGT (U/L)						
<45	61 (67.0)	38 (67.9)	1.000	37 (61.7)	21 (53.8)	0.573
≥45	30 (33.0)	18 (32.1)		23 (38.3)	18 (46.2)	
ALP (U/L)						
<125	75 (82.4)	44 (78.6)	0.719	48 (80.0)	30 (76.9)	0.909
≥125	16 (17.6)	12 (21.4)		12 (20.0)	9 (23.1)	
Alb (g/L)						
<35	19 (20.9)	16 (28.6)	0.388	15 (25.0)	9 (23.1)	1.000
≥35	72 (79.1)	40 (71.4)		45 (75.0)	30 (76.9)	
TIBL (µmol/L)						
<20.4	70 (76.9)	46 (82.1)	0.586	42 (70.0)	27 (69.2)	1.000
≥20.4	21 (23.1)	10 (17.9)		18 (30.0)	12 (30.8)	
CR (µmol/L)						
<84	89 (97.8)	55 (98.2)	1.000	58 (96.7)	36 (92.3)	0.618
≥84	2 (2.2)	1 (1.8)		2 (3.3)	3 (7.7)	
INR						
<1.15	84 (92.3)	52 (92.9)	1.000	55 (91.7)	34 (87.2)	0.702
≥1.15	7 (7.7)	4 (7.1)		5 (8.3)	5 (12.8)	
AFP (µg/mL)						
<400	43 (47.3)	29 (51.8)	0.716	34 (56.7)	22 (56.4)	1.000
≥400	48 (52.7)	27 (48.2)		26 (43.3)	17 (43.6)	
Tumor diameter (cm)	23.97 (4.05)	21.39 (6.72)	0.054	24.40 (3.56)	25.10 (3.80)	0.353
Tumor number						
Single tumor	32 (35.2)	21 (37.5)	0.913	24 (40.0)	14 (35.9)	0.843
Multiple tumors	59 (64.8)	35 (62.5)		36 (60.0)	25 (64.1)	

*AST, aspartate aminotransferase; ALT, alanine aminotransferase; GGT, gamma glutamyl transpeptidase; ALP, alkaline phosphatase; Alb, albumin; TIBL, total bilirubin; DIBL, direct bilirubin; CR, creatinine; INR, international normalized ratio; AFP, alpha fetoprotein; HBV, hepatitis B virus; HCV, hepatitis C virus; RFA, radiofrequency ablation; SR, surgical resection.*

**Figure 1:**
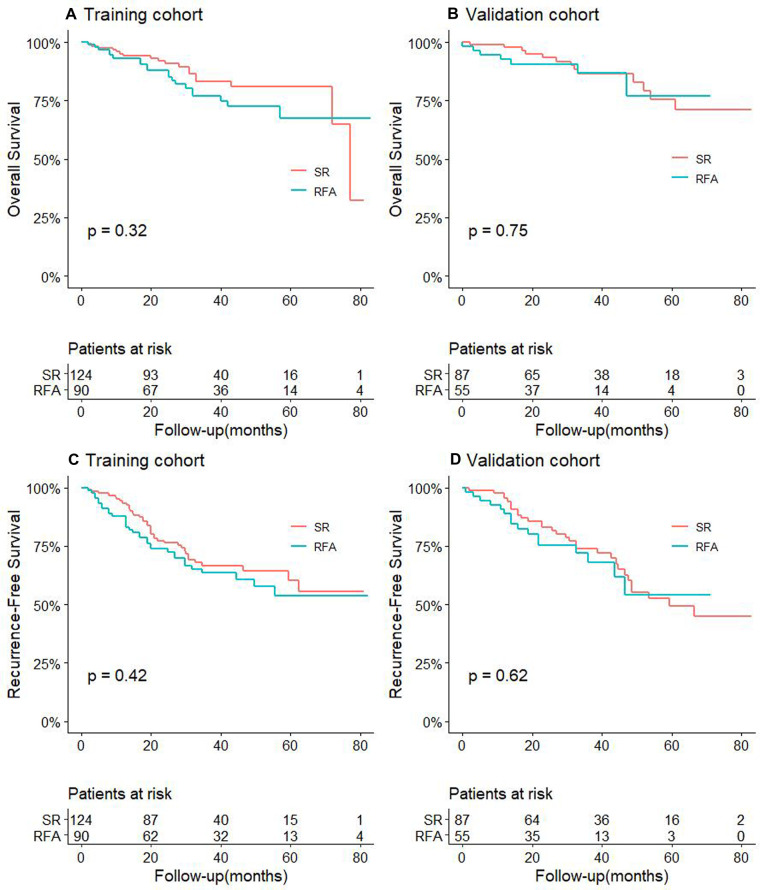
Comparison of OS between RFA and SR groups in training cohort (**A**) and validation cohort (**B**) for patients with CTC-negative; RFS comparison between RFA and SR groups in training cohort (**C**) and validation cohort (**D**) for patients with CTC-negative.

For CTC-negative patients, the 1-, 3-, and 5-year OS and RFS of RFA and SR in the training cohort and validation cohort are shown in Supplementary Table S2. And we found that there was no significant difference in the OS and the RFS between patients in the RFA group and those in the SR group, either in the training or validation cohort (*P *> 0.05, Figures 1A–D).

**Figure 2:**
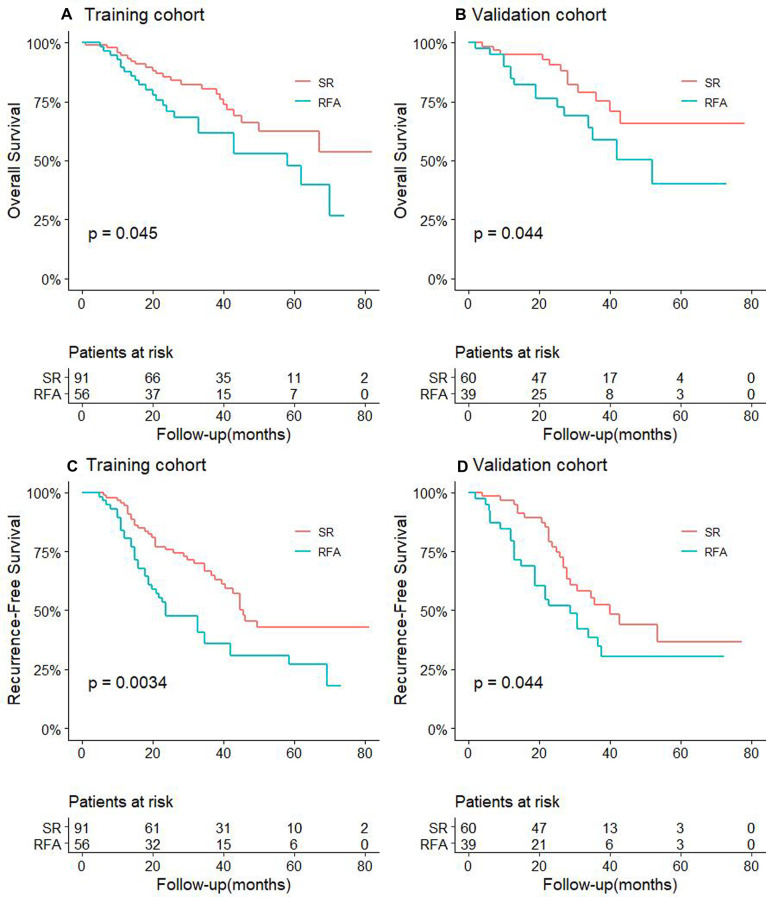
Comparison of OS between RFA and SR groups in training cohort (**A**) and validation cohort (**B**) for patients with CTC-positive; RFS comparison between RFA and SR groups in training cohort (**C**) and validation cohort (**D**) for patients with CTC-positive.

For CTC-positive patients, the 1-, 3-, and 5-year OS and RFS of RFA and SR in the training cohort and validation cohort also are shown in Supplementary Table S2. The results showed that in both the training cohort and the validation cohort, the OS and RFS of patients in the SR group were significantly longer than of those in the RFA, and the difference was statistically significant (*P *< 0.05, Figures 2A–D).

At the same time, we also performed univariate and multivariate Cox regression analyses on the related clinicopathological factors affecting OS and RFS in the CTC-positive population. We found that RFA was an independent risk factor for tumor recurrence and survival prognosis. Other factors such as AFP level ≥400 µg/L, tumor diameter ≥2 cm, and tumor number ≥2 were independent risk factors for postoperative recurrence and long-term survival (Tables 3, 4, *P *< 0.05). While Child-Pugh classification B and albumin <35 g/L were independent risk factors for the OS of patients (*P* < 0.05, Table 4).

**Table 3:** Univariate and multivariate analysis to identify independent risk factors of RFS in CTC-positive HCC.

Variable		Training Cohort (*n* = 147)		Validation cohort (*n* = 99)	
		Univariate Analysis		Univariate Analysis	
		HR (*CI*)	*P*	HR (*CI*)	*P*
Age	≥60 vs <60	0.826(0.594–1.148)	0.256	1.185(0.746–1.88)	0.472
Gender	Male vs female	1.323(0.915–1.915)	0.137	0.852(0.558–1.3)	0.457
HBV	Yes vs no	1.451(0.884–2.382)	0.141	0.958(0.496–1.848)	0.897
HCV	Yes vs no	1.208(0.592–2.467)	0.604	0.533(0.195–1.457)	0.220
Cirrhosis	Yes vs no	0.891(0.623–1.275)	0.529	0.807(0.515–1.267)	0.352
Child-Pugh Class	B vs A	0.681(0.385–1.203)	0.186	2.046(0.891–4.699)	0.092
ALT (U/L)	≥50 vs <50	0.775(0.497–1.21)	0.263	0.911(0.566–1.467)	0.701
AST (U/L)	≥40 vs <40	1.145(0.808–1.623)	0.446	1.343(0.902–1.999)	0.147
GGT (U/L)	≥60 vs <60	0.764(0.546–1.069)	0.116	1.51 (0.968–2.355)	0.069
ALP (U/L)	≥125 vs <125	1.266(0.868–1.845)	0.221	0.82 (0.491–1.368)	0.447
Alb (g/L)	≥35 vs <35	0.651(0.457–0.928)	0.018	0.946(0.603–1.483)	0.808
TIBL (µmol/L)	≥20.4 vs <20.4	0.808(0.538–1.213)	0.304	1.363(0.914–2.032)	0.129
CR (µmol/L)	≥104 vs <104	0.382(0.095–1.545)	0.177	1.74 (0.842–3.592)	0.135
INR	≥1.20 vs <1.20	0.864(0.479–1.56)	0.629	1.103(0.602–2.021)	0.750
AFP (µg/mL)	≥400 vs <400	1.593(1.15–2.205)	0.005	1.659 (1.1–2.504)	0.016
Tumor diameter (cm)	≥2 vs <2	2.604(1.745–3.886)	0.000	2.438(1.359–4.372)	0.003
Tumor number	Single vs Multiple	0.719(0.52–0.995)	0.046	0.507(0.339–0.76)	0.001
Therapeutic method	RFA vs SR	2.009(1.454–2.776)	0.000	1.892 (1.27–2.819)	0.002
**Variable**		**Multivariate Analysis**		**Multivariate Analysis**	
		**HR (*CI*)**	***P***	**HR (*CI*)**	***P***
Alb (g/L)	≥35 vs <35	0.836(0.797–1.791)	0.388		**NG**
AFP (µg/mL)	≥400 vs <400	1.792(1.239–2.591)	0.002	1.605(1.059–2.433)	0.026
Tumor diameter (cm)	≥2 vs <2	2.405(1.603–3.607)	0.000	2.623(1.457–4.724)	0.001
Tumor number	Single vs Multiple	0.647(0.462–0.905)	0.011	0.498 (0.33–0.749)	0.001
Therapeutic method	RFA vs SR	2.36 (1.669–3.337)	0.000	1.797(1.204–2.683)	0.004

*AST, aspartate aminotransferase; ALT, alanine aminotransferase; GGT, gamma glutamyl transpeptidase; ALP, alkaline phosphatase; Alb, albumin; TIBL, total bilirubin; DIBL, direct bilirubin; CR, creatinine; INR, international normalized ratio; AFP, alpha fetoprotein; HBV, hepatitis B virus; HCV, hepatitis C virus; RFA, radiofrequency ablation; SR, surgical resection.*

**Table 4:** Univariate and multivariate analysis to identify independent risk factors of OS in CTC-positive HCC.

Variable		Training Cohort (*n* = 147)		Validation cohort (*n* = 99)	
		Univariate Analysis		Univariate Analysis	
		HR (*CI*)	*P*	HR (*CI*)	*P*
Age	≥60 vs <60	1.042(0.678–1.603)	0.851	1.227(0.646–2.333)	0.532
Gender	Male vs female	0.783(0.511–1.202)	0.264	0.813(0.461–1.434)	0.475
HBV	Yes vs no	1.129(0.635–2.006)	0.680	1.323(0.477–3.671)	0.591
HCV	Yes vs no	0.491(0.121–1.997)	0.320	0.489(0.232–1.727)	0.997
Cirrhosis	Yes vs no	1.013(0.649–1.582)	0.953	1.207(0.607–2.401)	0.592
Child-Pugh Class	B vs A	5.2 (3.428–7.889)	0.000	0.678(0.093–4.931)	0.701
ALT (U/L)	≥50 vs <50	1.06 (0.631–1.781)	0.826	1.375(0.766–2.47)	0.286
AST (U/L)	≥40 vs <40	0.953(0.601–1.51)	0.837	1.71 (0.994–2.943)	0.053
GGT (U/L)	≥60 vs <60	0.754(0.491–1.159)	0.198	1.321(0.705–2.474)	0.385
ALP (U/L)	≥125 vs <125	1.537(0.972–2.429)	0.066	1.223(0.665–2.25)	0.518
Alb (g/L)	≥35 vs <35	0.18(0.117–0.275)	0.000	1.164(0.631–2.147)	0.628
TIBL (µmol/L)	≥20.4 vs <20.4	0.603(0.341–1.068)	0.083	1.672(0.971–2.879)	0.064
CR (µmol/L)	≥104 vs <104	1.28 (0.468–3.506)	0.630	0.781 (0.19–3.212)	0.732
INR	≥1.20 vs <1.20	1.163(0.602–2.249)	0.652	0.676(0.244–1.873)	0.452
AFP (µg/mL)	≥400 vs <400	3.024(1.917–4.77)	0.000	2.988(1.572–5.678)	0.001
Tumor diameter (cm)	≥2 vs <2	2.827(1.679–4.76)	0.000	2.984(1.276–6.977)	0.012
Tumor number	Single vs Multiple	0.581(0.382–0.884)	0.011	0.416(0.238–0.729)	0.002
Therapeutic method	RFA vs SR	1.9 (1.258–2.87)	0.002	3.488(1.943–6.263)	0.000
**Variable**		**Multivariate Analysis**		**Multivariate Analysis**	
		**HR (*CI*)**	***P***	**HR (*CI*)**	***P***
Child-Pugh Class	B vs A	1.813(1.026–3.202)	0.041	** **	**NG**
Alb (g/L)	≥35 vs <35	0.436(0.237–0.802)	0.008	** **	**NG**
AFP (µg/mL)	≥400 vs <400	1.875(1.101–3.193)	0.021	2.599(1.358–4.975)	0.004
Tumor diameter (cm)	≥2 vs <2	1.857(1.077–3.201)	0.026	2.966 (1.26–6.98)	0.013
Tumor number	Single vs Multiple	0.532(0.337–0.841)	0.007	0.437(0.248–0.772)	0.004
Therapeutic method	RFA vs SR	1.88 (1.192–2.965)	0.007	2.998(1.661–5.41)	0.000

*AST, aspartate aminotransferase; ALT, alanine aminotransferase; GGT, gamma glutamyl transpeptidase; ALP, alkaline phosphatase; Alb, albumin; TIBL, total bilirubin; DIBL, direct bilirubin; CR, creatinine; INR, international normalized ratio; AFP, alpha fetoprotein; HBV, hepatitis B virus; HCV, hepatitis C virus; RFA, radiofrequency ablation; SR, surgical resection.*

### RFA is Associated with Increased Early Recurrence in CTC-Positive Patients

**Figure 3:**
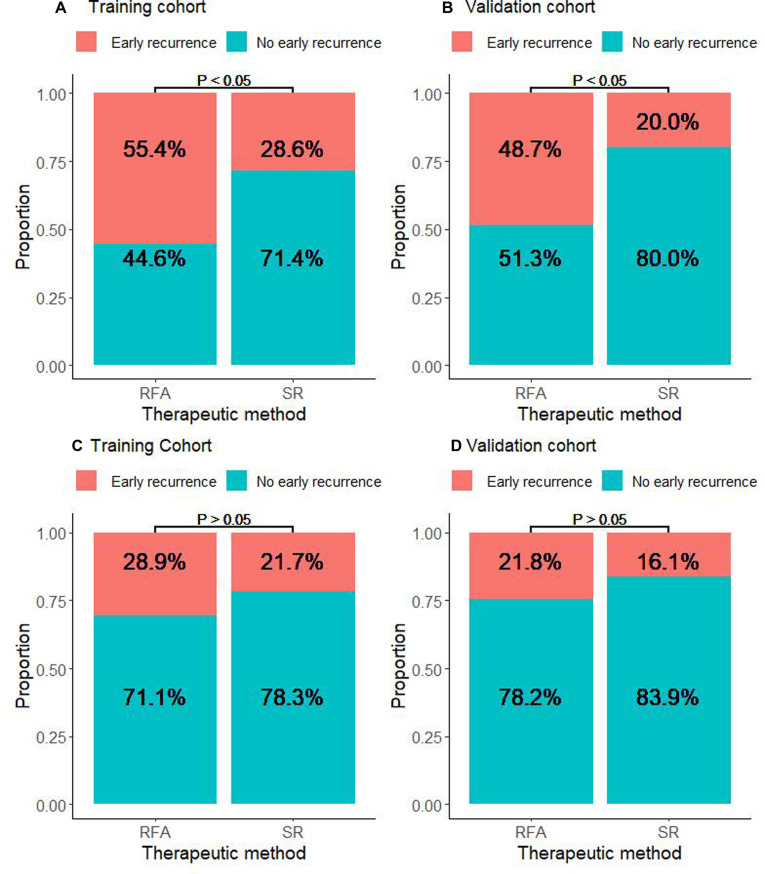
Association between RFA and early recurrence in HCC patients stratified by CTC. (**A**) Proportion of CTC-positive patients who experienced early or non-early recurrence in the RFA and SR groups in the training cohort. (**B**) Proportion of CTC-positive patients who experienced early or non-early recurrence in the RFA and SR groups in the validation cohort. (**C**) Proportion of CTC-negative patients who experienced early or non-early recurrence in the RFA and SR groups in the training cohort. (**D**) Proportion of CTC-negative patients who experienced early or non-early recurrence in the RFA and SR groups in the validation cohort.

**Figure 4:**
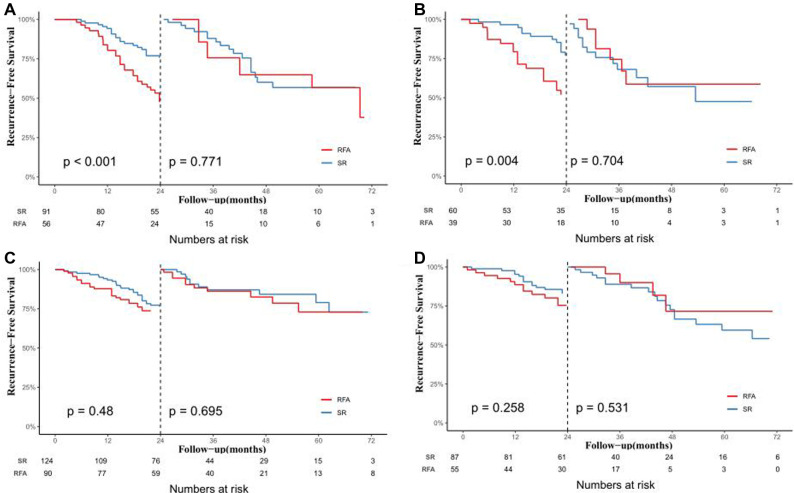
Analysis of the effect of different treatments on early and late postoperative recurrence in HCC patients by landmark method. In the training cohort (**A**) and validation cohort (**B**) of CTC-positive patients; In the training cohort (**C**) and validation cohort (**D**) of CTC-negative patients.

We further explored the CTCs status and postoperative recurrence patterns. Postoperative recurrence was divided into early recurrence and late recurrence with a threshold of 24 months. The results showed that for CTC-positive patients, the early recurrence rate of patients who underwent RFA in the training cohort was 55.4%. The early recurrence rate for patients in the SR group was 28.6%. The difference was statistically significant (*P* < 0.05, Figure 3A). At the same time, in the validation cohort, the early recurrence in the RFA group was significantly higher than that of the SR group. The early recurrence rates in the RFA group and SR group were 48.7% and 20%, respectively, which was statistically significant (*P *< 0.05, Figure 3B). For the CTC-negative population, in both the training cohort and the validation cohort, early recurrence was not related to the treatment method, and there was no statistical significance (*P* > 0.05, Figures 3C,D). Next, we also conducted a landmark analysis. The results showed that for CTC-positive patients, in the training cohort and the validation cohort, patients who received RFA were more likely to have an early recurrence than those who had SR, while there was no statistical difference in late recurrence rates (*P* < 0.05, Figures 4A,B). For CTC-negative patients, there was no relationship between recurrence pattern and treatment modality (*P* > 0.05, Figures 4C,D).

### CTCs Status Correlates with Microvascular Infiltration

For the population undergoing surgery, the CTC positive rates in the training cohort and validation cohort were 42.3% and 40.8%, respectively. The data showed that in the training cohort, CTC positivity was associated with AFP ≥ 400 µg/L, tumor number, and microvascular invasion (MVI) (*P *< 0.05, Table 5). At the same time, it was found that the clinical variables associated with CTC-positivity in the training group were tumor number and MVI (*P *<* *0.05, Table 5), but there was no significant correlation with other clinical variables, and there was no statistical significance. (*P* > 0.05, Table 5).

**Table 5:** Relationship between CTC status with clinical characteristics for patients in the surgery group.

Variable	Training cohort (*n* = 215)			Validation cohort (*n*= 147)		
	CTC = 0 (*n* = 124)	CTC≥ 1 (*n* = 91)	*P*	CTC = 0 (*n* = 87)	CTC≥ 1 (*n* = 60)	*P*
Age (years)						
<60	42 (33.9)	33 (36.3)	0.827	36 (41.4)	15 (25.0)	0.061
≥60	82 (66.1)	58 (63.7)		51 (58.6)	45 (75.0)	
Gender						
Female	37 (29.8)	27 (29.7)	1.000	24 (27.6)	14 (23.3)	0.699
Male	87 (70.2)	64 (70.3)		63 (72.4)	46 (76.7)	
HBV						
No	9 (7.3)	10 (11.0)	0.478	11 (12.6)	4 (6.7)	0.368
Yes	115 (92.7)	81 (89.0)		76 (87.4)	56 (93.3)	
HCV						
No	122 (98.4)	86 (94.5)	0.232	84 (96.6)	57 (95.0)	0.965
Yes	2 (1.6)	5 (5.5)		3 (3.4)	3 (5.0)	
Cirrhosis						
No	30 (24.2)	27 (29.7)	0.458	23 (26.4)	16 (26.7)	1.000
Yes	94 (75.8)	64 (70.3)		64 (73.6)	44 (73.3)	
Child-Pugh						
A	114 (91.9)	78 (85.7)	0.217	80 (92.0)	53 (88.3)	0.653
B	10 (8.1)	13 (14.3)		7 (8.0)	7 (11.7)	
ALT (U/L)						
<50	100 (80.6)	78 (85.7)	0.429	72 (82.8)	45 (75.0)	0.348
≥50	24 (19.4)	13 (14.3)		15 (17.2)	15 (25.0)	
AST (U/L)						
<40	85 (68.5)	68 (74.7)	0.403	59 (67.8)	44 (73.3)	0.593
≥40	39 (31.5)	23 (25.3)		28 (32.2)	16 (26.7)	
GGT (U/L)						
<45	88 (71.0)	61 (67.0)	0.64	66 (75.9)	37 (61.7)	0.096
≥45	36 (29.0)	30 (33.0)		21 (24.1)	23 (38.3)	
ALP (U/L)						
<125	110 (88.7)	75 (82.4)	0.264	74 (85.1)	48 (80.0)	0.563
≥125	14 (11.3)	16 (17.6)		13 (14.9)	12 (20.0)	
Alb (g/L)						
<35	17 (13.7)	19 (20.9)	0.228	12 (13.8)	15 (25.0)	0.132
≥35	107 (86.3)	72 (79.1)		75 (86.2)	45 (75.0)	
TIBL (µmol/L)						
<20.4	96 (77.4)	70 (76.9)	1.000	74 (85.1)	42 (70.0)	0.056
≥20.4	28 (22.6)	21 (23.1)		13 (14.9)	18 (30.0)	
CR (µmol/L)						
<84	122 (98.4)	89 (97.8)	1.000	83 (95.4)	58 (96.7)	1.000
≥84	2 (1.6)	2 (2.2)		4 (4.6)	2 (3.3)	
INR						
<1.15	117 (94.4)	84 (92.3)	0.748	83 (95.4)	55 (91.7)	0.563
≥1.15	7 (5.6)	7 (7.7)		4 (4.6)	5 (8.3)	
AFP (µg/mL)						
<400	99 (79.8)	43 (47.3)	<0.001	62 (71.3)	34 (56.7)	0.099
≥400	25 (20.2)	48 (52.7)		25 (28.7)	26 (43.3)	
Tumor diameter (cm)	22.92 (4.87)	23.97 (4.05)	0.096	23.40 (4.64)	24.40 (3.56)	0.163
Tumor number						
Single tumor	72 (58.1)	32 (35.2)	0.001	51 (58.6)	24 (40.0)	0.040
Multiple tumors	52 (41.9)	59 (64.8)		36 (41.4)	36 (60.0)	
Grade						
I + II	105 (84.7)	73 (80.2)	0.501	74 (85.1)	48 (80.0)	0.563
III + IV	19 (15.3)	18 (19.8)		13 (14.9)	12 (20.0)	
MVI						
No	104 (83.9)	63 (69.2)	0.017	73 (83.9)	41 (68.3)	0.043
Yes	20 (16.1)	28 (30.8)		14 (16.1)	19 (31.7)	

*AST, aspartate aminotransferase; ALT, alanine aminotransferase; GGT, gamma glutamyl transpeptidase; ALP, alkaline phosphatase; Alb, albumin; TIBL, total bilirubin; DIBL, direct bilirubin; CR, creatinine; INR, international normalized ratio; AFP, alpha fetoprotein; HBV, hepatitis B virus; HCV, hepatitis C virus; RFA, radiofrequency ablation; SR, surgical resection.*

In addition, we also used univariate and multivariate logistic regression to explore the factors associated with MVI. The results showed that positive CTC, the level of AFP ≥ 400 µg/L, and tumor number were independent predictors of MVI in both the training and validation cohorts (*P *< 0.05, Table 6).

**Table 6:** Univariate and multivariate analysis to predict independent risk factors for microvascular invasion.

Variable		Training Cohort (*n* = 215)		Validation cohort (*n* = 147)	
		Univariate Analysis		Univariate Analysis	
		OR (*CI*)	*P*	OR (*CI*)	*P*
Age	≥60 vs <60	1.51 (0.74–3.07)	0.256	0.63 (0.28–1.4)	0.257
Gender	Male vs. female	1.04 (0.51–2.1)	0.918	0.91 (0.38–2.18)	0.832
HBV	Yes vs. no	0.43 (0.13–1.39)	0.159	0.6 (0.24–1.48)	0.270
HCV	Yes vs. no	1.5 (0.5–4.5)	0.467	3.61 (0.49–26.69)	0.208
Cirrhosis	Yes vs. no	0.69 (0.25–1.89)	0.471	0.59 (0.25–1.38)	0.223
Child-Pugh Class	B vs A	1.52 (0.38–6.13)	0.553	3.21 (0.91–11.3)	0.069
ALT (U/L)	≥50 vs <50	0.9 (0.4–2.04)	0.806	0.47 (0.15–1.45)	0.188
AST (U/L)	≥40 vs <40	0.66 (0.32–1.37)	0.265	0.56 (0.22–1.41)	0.218
GGT (U/L)	≥60 vs <60	1.03 (0.52–2.07)	0.925	1.46 (0.65–3.32)	0.361
ALP (U/L)	≥125 vs <125	1.69 (0.61–4.72)	0.315	2.11 (0.81–5.54)	0.128
Alb (g/L)	≥35 vs <35	0.59 (0.3–1.18)	0.137	0.55 (0.25–1.22)	0.143
TIBL (µmol/L)	≥20.4 vs <20.4	0.89 (0.45–1.76)	0.74	0.44 (0.14–1.38)	0.160
CR (µmol/L)	≥104 vs <104	1.83 (0.8–4.21)	0.155	1.61 (0.46–5.6)	0.455
INR	≥1.20 vs <1.20	1.96 (0.87–4.4)	0.103	2.29 (0.82–6.39)	0.114
AFP (µg/mL)	≥400 vs <400	4.95 (2.45–9.97)	<0.001	4.59 (1.84–11.43)	0.001
Tumor diameter (cm)	≥2 vs <2	2.66 (1.17–6.06)	0.020	1.61 (0.6–4.27)	0.342
Tumor number	Single vs Multiple	0.36 (0.18–0.7)	0.003	0.14 (0.06–0.38)	<0.001
Grade	III + IV vs I + II	1.81 (0.87–3.77)	0.115	5.52 (2.2–13.86)	<0.001
CTCs status	Positive vs Negative	5.08 (2.52–10.25)	<0.001	2.94 (1.33–6.51)	0.008
**Variable**		**Multivariate Analysis**		**Multivariate Analysis**	
		**OR (*CI*)**	***P***	**OR (*CI*)**	***P***
AFP (µg/mL)	≥400 vs <400	2.93 (1.32–6.5)	0.041	4.78 (1.75–13.06)	0.002
Tumor diameter (cm)	≥2 vs <2	2.580 (1.06–6.26)	0.008	**NG**	**NG**
Tumor number	Single vs Multiple	0.44 (0.21–0.92)	0.021	0.18 (0.07–0.52)	0.001
Grade	III + IV vs I + II	**NG**	**NG**	3.89 (0.98–11.8)	0.057
CTCs status	Positive vs Negative	3.26 (1.47–7.24)	0.007	2.65 (1.04–6.73)	0.041

*AST, aspartate aminotransferase; ALT, alanine aminotransferase; GGT, gamma glutamyl transpeptidase; ALP, alkaline phosphatase; Alb, albumin; TIBL, total bilirubin; DIBL, direct bilirubin; CR, creatinine; INR, international normalized ratio; AFP, alpha fetoprotein; HBV, hepatitis B virus; HCV, hepatitis C virus; RFA, radiofrequency ablation; SR, surgical resection; CTC, circulating tumor cells.*

## Discussion

The treatment mode of HCC has developed from the single surgical resection mode to the multidisciplinary team (MDT) mode in the past decades. The multidisciplinary participation model significantly improves the survival time of HCC patients, but its overall effect is still not ideal, mainly because HCC patients are prone to postoperative recurrence.

Since Rossi et al. firstly used RFA to treat HCC (23), RFA has become one of the important components in the comprehensive treatment of HCC. The current clinical application of RFA mainly depends on the experience of traditional tumor characterization (24). However, there is still controversy about whether to choose RFA or SR for the treatment of small hepatocellular carcinoma (SHCC, diameter≤3) (9–13, 25–28). A retrospective clinical study conducted by Peng et al. found that in the SHCC population, the RFS and OS of patients who were selected for RFA and patients with SR were similar, and the difference was not statistically significant (10). Furthermore, another retrospective study from Italy Livraghi, T. et al also found that the efficacy of RFA is completely comparable to SR, and they should give priority to RFA rather than SR for patients with SHCC, because RFA has many advantages including less trauma, lower cost, and fewer complications (25). In contrast, a meta-analysis by Dong W et al. showed that the SR group had a better survival prognosis than non-surgical ablation (26). and another research team from China also investigated this issue. In their study, which enrolled 605 patients, RFA was more prone to postoperative recurrence than SR in both Barcelona stage 0∼A (27). Why do similar studies have different results? To answer this question, we should explore the answer from the perspective of which factors affect the efficacy of radiofrequency ablation.

In previous studies, it was found that there are many clinical factors affecting the efficacy of RFA, such as tumor diameter, and tumor number (29–31), among which the most striking is whether the tumor is accompanied by MVI (7). Because tumor diameter and tumor number can be assessed by preoperative imaging examinations, complete ablation of the main lesion can be achieved by increasing the scope of RFA. However, from a pathological point of view, HCC lesions do not only include the main tumor but also the surrounding MVI and satellite nodules (32). Unfortunately, current imaging and serological tests cannot accurately assess MVI and satellite nodules preoperatively. This leads to the fact that RFA can only guarantee the quality of ablation of the main lesions, but cannot determine whether the occult lesions are effectively removed, which may be the reason for the early recurrence after RFA (33, 34). Therefore, we urgently need a new and reliable indicator to assess whether these occult lesions exist in HCC patients, and to guide the application of RFA in SHCC.

CTCs are malignant tumor cells that invade into the peripheral blood via the form of Epithelial-Mesenchymal Transition (EMT), which can serve as a prognostic indicator of disease progression and it is associated with the aggressiveness of tumors (35–37). CTCs are often used for the prognostic monitoring of breast, colorectal, and prostate cancers (14, 21, 38–41). Recently, more and more studies have confirmed that CTC detection is considered a reliable indicator of early screening for cancer, postoperative recurrence or metastasis surveillance in HCC patients (42–45). In particular, CTC status is also highly correlated with MVI which affects the efficacy of RFA (7, 46). Our study also revealed that CTCs status was an independent predictor of whether HCC was associated with MVI. In other words, patients with positive CTCs are more likely to be accompanied by MVI. Besides this, some literature has confirmed that CTCs are more accurate in predicting MVI than AFP and other pathological factors (46–48). Moreover, preoperative CTCs status could also reflect the severity of MVI, as CTC status positively correlated with the number and distance of MVI in peritumoral tissues (49). In summary, we have reason to believe that the preoperative detection of CTCs can indirectly determine whether the patient has MVI, to achieve the purpose of precise treatment.

In this study. We explored whether CTC status could affect the efficacy of RFA. Our data illustrated that for CTC-positive patients, the RFS and OS of the RFA group are inferior to the SR group in both the training and validation cohorts. Moreover, we have become conscious of the fact that RFA is associated with early recurrence, but not late recurrence. In short, RFA affected the OS of CTC-positive HCC patients mainly achieved by increasing the risk of early recurrence, as the probability of late recurrence was similar to that of patients who underwent SR. The conclusion is also verified in the validation cohort. It is well known that early postoperative recurrence of HCC is usually caused by residual occult lesions (50). Considering that the safety margin recommended by the current clinical guidelines for RFA is much lower than that of surgical resection (17), and for high-risk patients with preoperatively predicted MVI, RFA will greatly increase the probability of residual occult lesions compared with SR (7), which is also consistent with our findings.

Apart from that, our studies also reveal that the OS and RFS of CTC-negative patients in the RFA group and the SR group were roughly the same, and RFA did not increase the risk of early postoperative recurrence, likely due to the intrinsic low risk of recurrence in CTC-negative patients. Thus, preoperative CTCs detection has a high clinical guiding value, which might help to evaluate whether RFA or SR should be chosen for patients with SHCC. For patients with positive CTCs, SR should be the preferred treatment, or for patients who are only willing to receive RFA, postoperative adjuvant TACE may be recommended to improve the survival and prognosis of patients (50).

Our study possesses several limitations. 1. This study is a retrospective cohort study, there are many potential biases and confounding factors that cannot be eliminated, and the conclusions of the study need to be confirmed by a prospective study with a larger sample size. 2. Our research population is mainly the Chinese population. The vast majority of patients have hepatitis B virus-related hepatocellular carcinoma. Whether the conclusion is suitable for the hepatitis C and alcohol-related HCC population is still unknown. 3. There may be temporal heterogeneity in the detection of CTCs, and the number of CTCs in the peripheral blood of tumors in different periods may be different (51). The conclusion of this study may only be suitable for HCC patients whose tumor diameters are less than or equal to 3 cm. 4. The cost of CTCs detection is high, and it cannot be performed as a routine test in some areas with poor economic conditions, but we believe that with the improvement of detection methods, the cost of detection can be reduced to be more commonly used in clinical practice.

## Conclusion

This study provides a new indicator for deciding whether to choose RFA or SR in the treatment of small hepatocellular carcinoma (SHCC). RFA should be avoided as a priority treatment for SHCC patients with positive preoperative CTCs.

## Data Availability

The original contributions presented in the study are included in the article/**Supplementary Material**, further inquiries can be directed to the corresponding author/s.
