# Supplementary figures and images for: Circulating Tumor Cells as an Indicator of Treatment Options for Hepatocellular Carcinoma Less Than or Equal to 3 cm in Size: A Multi-Center, Retrospective Study
Source: Front Surg. 2022 Jun 20;9:895426. doi: 10.3389/fsurg.2022.895426 (PMC9251203; doi:10.3389/fsurg.2022.895426)

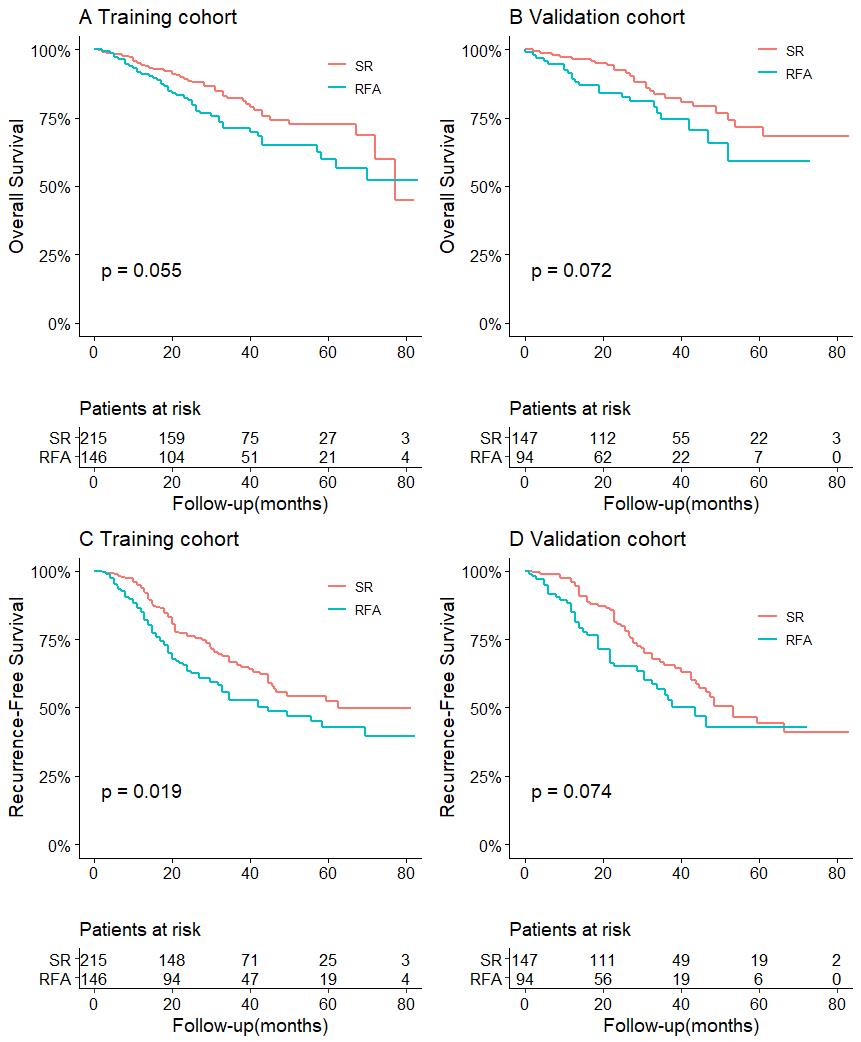

Supplement: Supplementary file 1 [file Image_1_v1.jpeg]
